# Blood-Based Biomarkers for Post-Stroke Cognitive Impairment

**DOI:** 10.3390/cimb48070702

**Published:** 2026-07-10

**Authors:** Jie Pu, Wang Guo, Hongxin Li, Zhihao Wang, Tangda Mu, Fuli Xie, Jie Chen, Jiawei Cao, Chengfei Zhong, Hongyu Li, Qiang Tang

**Affiliations:** 1 Graduate School of Heilongjiang University of Chinese Medicine, Harbin 150040, China; 15086928363@163.com (J.P.); guowang199204@163.com (W.G.); 13953749130@163.com (H.L.); wangzhihao698@163.com (Z.W.); 17341021482@163.com (T.M.); 18280861302@163.com (F.X.); 13072606616@163.com (J.C.); c2023447091@163.com (J.C.); 17865659116@163.com (C.Z.); 2 Heilongjiang University of Chinese Medicine Affiliated Second Hospital, Harbin 150001, China

**Keywords:** post-stroke cognitive impairment, blood-based testing, biomarkers, clinical diagnosis

## Abstract

Stroke remains the second leading cause of death and the third leading cause of death and disability. With the growing incidence of strokes, the incidence of post-stroke cognitive impairment (PSCI) among patients is on a steady rise. These patients commonly present with impairments in memory, attention, executive function, comprehension, and judgment, substantially affecting their everyday lives. Magnetic resonance image (MRI) and cognitive assessment scales are widely employed as diagnostic tools for PSCI. They also have notable limitations. Furthermore, cerebrospinal fluid collection is relatively complex. Growing evidence suggests that blood-based biomarkers are closely involved in the onset and progression of PSCI, as bioassay techniques and equipment have become increasingly popular and optimized. Therefore, blood-based biomarkers play an essential role in the pathogenesis and progression of PSCI. This review concentrates on the research progress of blood-based biomarkers in PSCI to explore their significance for disease identification, with the aim of offering novel insights into the clinical diagnosis and therapy of PSCI.

## 1. Introduction

**Figure 1:**
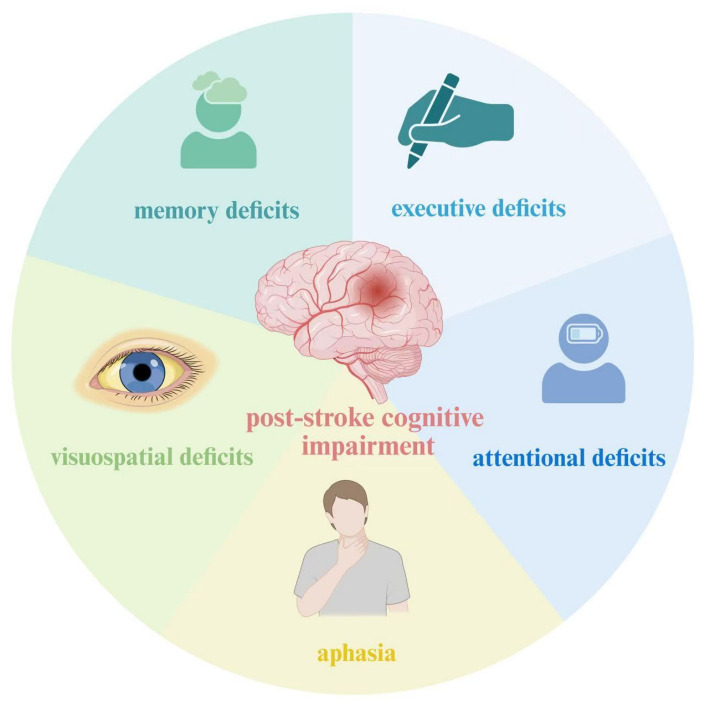
Clinical manifestations of PSCI. *Created in BioRender. Pu, J. (2026). BioRender.com/cgr7vx4*.

Stroke remains the second leading cause of death and the third leading cause of death and disability combined in the world. The Global Burden of Disease (GBD) indicates that the number of individuals who experience a stroke, die from it, or live with a disability as a result of a stroke has risen substantially worldwide between 1990 and 2021 [1]. The estimated global cost of stroke is over US$890 billion (0.66% of the global GDP) per year, and is projected to almost double by 2050 [2]. Incident stroke was associated with acute decline in cognitive function and accelerated and persistent cognitive decline over 6 years [3]. As the incidence of stroke continues to rise, the incidence of post-stroke cognitive impairment (PSCI) among patients is on a steady rise. PSCI represents a group of common complications that occur following stroke and a spectrum of cognitive dysfunction occurring after stroke, ranging from early cognitive changes during the acute and sub-acute phases to persistent cognitive impairment and post-stroke dementia during long-term follow-up. Clinical manifestations include aphasia, memory, visuospatial, attentional, and executive deficits (Figure 1) and occur in approximately one-third of stroke survivors [4]. Within three months after stroke, 24–39% of patients in Europe experience cognitive impairment, compared with as high as 69.8% in Asia. These patients commonly present with impairments in memory, attention, executive function, comprehension, and judgment, substantially affecting their everyday lives [5]. At present, magnetic resonance image (MRI) and cognitive assessment scales are widely employed as diagnostic tools for PSCI; however, they also have notable limitations. Imaging assessments are expensive, and MRI may fail to capture functional alterations in patients who do not exhibit clear structural brain changes [6]. There are several limitations associated with cognitive assessment scales, and the observed heterogeneity may stem from variations in the tools employed for cognitive evaluation [7]. At present, no agreement has been reached regarding the optimal cognitive assessment scale for screening PSCI [8]. Even within the same assessment tool, multiple versions are available, and the optimal cutoff thresholds may differ depending on the stage of the disease [9]. They are also susceptible to influences from factors such as culture, language, education, and age [10,11]. Cognitive assessment is inherently subjective, and in clinical practice, evaluations may vary between examiners. Furthermore, the collection of cerebrospinal fluid is technically challenging, especially in individuals with stroke, highlighting the importance of developing blood-based biomarkers as a major direction for future PSCI studies [12]. Given their ease of access, minimal invasiveness, objectivity, and cost efficiency, blood-based biomarkers hold great promise.

Growing evidence suggests that blood-based biomarkers are closely involved in the onset and progression of PSCI. In this review, we summarize recent progress in this rapidly advancing area of blood biomarker research for PSCI. The main findings of the reviewed papers are discussed in the context of a series of pathological mechanisms implicated in PSCI. We aim to outline the current state of the field, identify key limitations in the existing literature, and propose future directions to improve understanding of the diagnostic value of blood-based biomarkers in PSCI, thereby facilitating early detection and enhancing the effectiveness of clinical management.

## 2. Search Method

We searched the PubMed, Web of Science, Embase databases for articles related to this review and manually identified and included eligible references. We use the following keywords to search for all possible combinations: “stroke,” “post-stroke cognitive impairment,” “cognitive impairment,” “blood-based testing,” and “biomarker.”

## 3. Pathophysiology of PSCI

**Figure 2:**
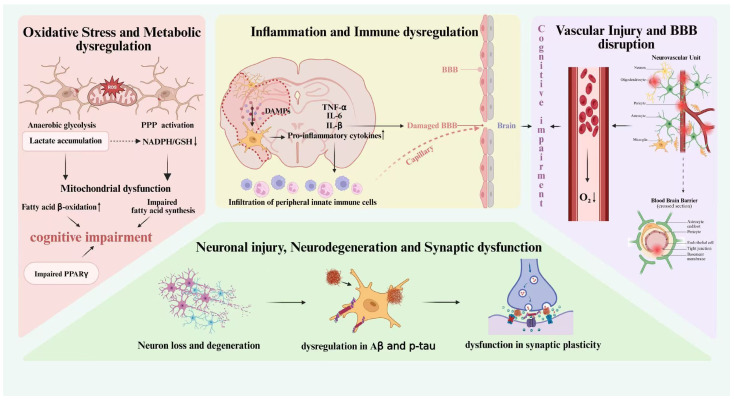
Pathophysiological mechanisms of post-stroke cognitive impairment (PSCI). *Created in BioRender. Pu, J. (2026). BioRender.com/5xlx4f*.

The development of post-stroke cognitive impairment involves a dynamic and interconnected pathological cascade rather than a consequence of a single pathological event. Following acute ischemic injury, energy failure and cellular damage trigger the release of damage-associated molecular patterns (DAMPs), which activate systemic and central inflammatory responses. Neuroinflammation and oxidative stress subsequently act as key amplifying mechanisms, in which excessive reactive oxygen species production further aggravates neuronal injury, endothelial dysfunction, and tissue damage. Meanwhile, disruption of the blood–brain barrier (BBB) increases vascular permeability, facilitating the infiltration of peripheral immune cells and neurotoxic molecules into the brain, thereby further promoting secondary neuronal damage and impaired neural homeostasis. Over time, persistent inflammatory activation, oxidative imbalance, and vascular dysfunction contribute to delayed neurodegeneration, impaired synaptic plasticity, and disruption of neural network integration. In addition, disturbances in excitatory and inhibitory neurotransmitter balance may further compromise synaptic function and cognitive processing, ultimately leading to persistent cognitive decline after stroke. Accordingly, different classes of blood-based biomarkers may reflect distinct stages of this pathological continuum, ranging from early inflammatory and vascular responses to delayed neuronal degeneration and long-term cognitive deterioration [13] (Figure 2).

### 3.1. Inflammation and Immune Dysregulation

Inflammation plays a central role in the pathological processes after stroke, and a persistent pro-inflammatory milieu is considered a major driver of long-term cognitive decline [14], and includes activation of resident inflammatory cells, followed by the release of inflammatory mediators, and the recruitment and migration of leukocytes through the blood–brain barrier (BBB) [15]. During the acute stage of cerebral ischemia, hypoxia-driven activation of glial cells induces the release of pro-inflammatory factors, such as TNF-α and various interleukins. These factors not only intensify the local inflammatory milieu but also have the potential to promote remote neuronal injury and trigger apoptotic processes [16]. Evidence indicates that TNF-α is significantly elevated in the early post-stroke period, where it plays a pivotal role in driving microglial activation and perpetuating inflammatory signaling cascades [17]. Meanwhile, endothelial nitric oxide synthase impairment exacerbates ROS production through diminished nitric oxide (NO) synthesis, while elevated ROS levels, in turn, induce the expression of pro-inflammatory genes [18]. Disruption of the equilibrium between pro-inflammatory and anti-inflammatory cytokines within the brain triggers activation of cerebral microvasculature and impairs BBB function, thereby worsening neurological outcomes and eventually resulting in cognitive decline and dementia [19]. Evidence suggests that immune response during the early stage of stroke is strongly linked to long-term cognitive performance [20]. Experimental and clinical studies have suggested that autoimmune mechanisms may contribute to the development of post-stroke cognitive decline [21]. In the setting of acute cerebral ischemia, damage-associated molecular patterns (DAMPs) are liberated, initiating activation of brain-resident microglia and facilitating the influx of peripheral innate immune cells, ultimately intensifying ischemic damage [22]. Concurrently, ischemic injury in the brain generates harmful substances, especially debris from necrotic cells, which activates immune-mediated inflammatory cascades, contributing to subsequent repair processes and tissue damage [23].

Therefore, immune-mediated inflammatory processes are key contributors to the development of PSCI, while neuroinflammatory activity shows a strong connection with mitochondrial impairment and oxidative stress [24]. Increasing evidence suggests that heightened systemic inflammation, especially involving IL-1β and TNF-α, aggravates oxidative stress in the nervous system, leading to neuronal damage and eventual impairment of cognitive function [25].

### 3.2. Oxidative Stress and Metabolic Dysregulation

During the early phase after stroke, excessive production of reactive oxygen species (ROS), mainly driven by NADPH oxidase (NOX) activation, mitochondrial dysfunction, and impaired antioxidant capacity, represents a critical mechanism underlying ischemic brain injury. Persistent oxidative imbalance not only induces lipid peroxidation and neuronal damage but also interacts with inflammatory signaling pathways, endothelial dysfunction, and blood–brain barrier (BBB) disruption, thereby amplifying secondary brain injury. Importantly, endogenous antioxidant defense systems, including the nuclear factor erythroid 2-related factor 2 (Nrf2) pathway and thioredoxin-dependent redox regulation, play essential roles in maintaining cellular redox homeostasis and promoting neuronal survival after stroke. Dysregulation of these protective mechanisms may contribute to prolonged oxidative injury and impaired neural network remodeling, ultimately facilitating cognitive decline after stroke [26]. Moreover, oxidative stress may impair the function of peroxisome proliferator-activated receptor-γ (PPAR-γ) and matrix metalloproteinases, which may contribute to white matter damage linked to cognitive impairment [27]. Overall, the evidence presented suggests that oxidative stress plays an essential role in the development of PSCI.

Excessive production of reactive oxygen species (ROS), mainly driven by NADPH oxidase (NOX) activation, mitochondrial dysfunction, and impaired antioxidant capacity, represents a critical mechanism underlying ischemic brain injury. Persistent oxidative imbalance not only induces lipid peroxidation and neuronal damage but also interacts with inflammatory signaling pathways, endothelial dysfunction, and blood–brain barrier (BBB) disruption, thereby amplifying secondary brain injury. Importantly, endogenous antioxidant defense systems, including the nuclear factor erythroid 2-related factor 2 (Nrf2) pathway and thioredoxin-dependent redox regulation, play essential roles in maintaining cellular redox homeostasis and promoting neuronal survival after stroke. Dysregulation of these protective mechanisms may contribute to prolonged oxidative injury and impaired neural network remodeling, ultimately facilitating cognitive decline after stroke.

The existing research indicates that metabolic disturbances exert complex and multifaceted effects in PSCI. Following ischemic stroke (IS), impaired cerebral blood flow limits the supply of oxygen and glucose to brain tissue, thereby forcing glucose metabolism toward anaerobic metabolism. This shift is accompanied by activation of the pentose phosphate pathway (PPP), which disturbs the homeostatic balance within the brain environment and contributes to cognitive decline. Dysregulation of lipid metabolism is likewise prominent following IS. In normal physiological states, fatty acids are processed by synthetic pathways and β-oxidation. However, after IS, decreased production of ATP and acetyl-CoA, suppression of the tricarboxylic acid cycle, and activation of the hypothalamic–pituitary–adrenal (HPA) axis synergistically suppress fatty acid biosynthesis. In contrast, β-oxidation of fatty acids is upregulated following IS, generating considerable energy that facilitates brain tissue recovery. Nevertheless, decreased total fatty acid content and hyperactivation of β-oxidation could promote oxidative stress, worsening brain tissue damage as well as ultimately leading to PSCI. Following ischemic stroke, endoplasmic reticulum (ER) stress in brain tissue quickly triggers protein misfolding through activation of the unfolded protein response (UPR), thereby enhancing degradation of protein. In addition, the reduced activity of eIF2α and TORC1 suppresses protein synthesis and accelerates protein breakdown a step further. The metabolic imbalance finally disrupts the equilibrium between protein synthesis and degradation, contributing to cognitive impairment [28].

### 3.3. Vascular Injury and Blood–Brain Barrier Disruption

A consequence of neuroinflammatory response is perturbation of the cerebrovascular system [29]; at its essence, stroke is primarily a vascular disorder [30]. Evidence from experimental animal models suggests that oxidative stress contributes to the development of blood–brain barrier (BBB) impairment in stroke-prone spontaneously hypertensive rats [31]. Acute stroke-induced injury eventually disrupts neurovascular unit (NVU) function, which represents the smallest building block of brain parenchyma and plays a central role in the development of most brain dysfunctions [32]. The NVU consists of various cell populations such as neurons, glia, and vascular cells [33], which work together to support the NVU’s critical functions in cerebral blood flow regulation and in preserving the homeostasis of the brain parenchymal milieu, serving as a major structural component of the BBB [34]. BBB dysfunction is thought to play a role in the development of cognitive impairment. Following stroke, BBB integrity is compromised, resulting in the indiscriminate leakage of blood components into the brain [35]. A growing body of evidence indicates that capillary dysfunction should also be considered an important mechanism in cerebrovascular disease, by impairing oxygen extraction rather than limiting blood supply [36]. Evidence suggests that capillary dysfunction plays a role in post-stroke tissue damage and is also involved in cognitive decline in vascular dementia [37]. In addition to vessel occlusion, cerebral ischemia and reperfusion induce a complex cascade of physiological responses involving pronounced neuroinflammatory and neurodegenerative processes that are not addressed by revascularization therapy, potentially contributing to limited functional recovery [38].

### 3.4. Neuronal Injury, Neurodegeneration and Synaptic Dysfunction

A growing body of evidence indicates that neuronal degeneration may emerge in brain regions remote from the primary lesion weeks to months after stroke. This process, known as secondary neurodegeneration (SND), is characterized by the gradual death of neurons in anatomically connected distal areas that were not exposed to the initial reduction in cerebral blood flow [39]. Current evidence suggests that the thalamus, hippocampus, and basal ganglia are among the regions most susceptible to such effects [40]. A comparable study conducted 15 months after stroke reported that hippocampal degeneration was closely associated with deficits in studying, memory, as well as overall cognitive function in patients with post-stroke cognitive impairment [41]. In addition, emerging evidence suggests that post-stroke atrophy in the thalamus and hippocampus progress more rapidly than in Alzheimer’s disease (AD), implying an accelerated degenerative process following stroke and reinforcing the link between SND and the onset of cognitive impairment [42]. Evidence suggests that regions affected by SND share common pathophysiological characteristics with other neurodegenerative disorders, such as dysregulation in amyloid-beta (Aβ) [43] and hyperphosphorylated tau [44]. Experimental animal models of cognitive impairment have also revealed the accumulation of amyloid precursor protein within areas of ischemic injury [45]. Recent evidence indicates that glial activation and the release of pro-inflammatory cytokines promote accumulation of Aβ and hyperphosphorylated tau in stroke pathology [46]. Reduced resting cerebral blood flow, together with the buildup of neurotoxic plasma proteins, can promote neuronal damage and neurodegeneration, even without the presence of Aβ. Furthermore, dysfunction of the NVU exacerbates Aβ buildup by impairing its clearance and enhancing its production [47]. The Aβ-independent and Aβ-dependent pathways are thought to act synergistically in driving cognitive impairment [32]. Post-stroke deficits in synaptic plasticity may lead to the development of PSCI. Given its critical role in cognitive recovery, enhanced synaptic efficacy and more efficient synaptic transmission enhance the capacity for information processing and storage in the central nervous system, ultimately promoting cognitive performance [48]. Following stroke, ischemia and hypoxia induce brain tissue damage, prompting a rapid release of pro-inflammatory factors such as TNF-α and IL-1β. These responses intensify local inflammation, and their sustained elevation promotes neuronal dysfunction, disturbs neurotransmitter balance, and compromises synaptic plasticity, culminating in cognitive impairments [49,50].

## 4. Blood-Based Biomarkers in PSCI

Following stroke onset, cognitive decline does not result from a single pathological event but rather from a dynamic and interconnected cascade involving neuroinflammation, oxidative stress, vascular dysfunction, neuronal injury, and impaired neural repair [13]. Therefore, circulating biomarkers associated with PSCI should not be interpreted as isolated molecular signals, but rather as peripheral indicators reflecting different biological processes involved in disease development and progression. The following sections summarize currently investigated blood-based biomarkers according to their predominant biological pathways.

### 4.1. Inflammation and Immune Biomarkers

#### 4.1.1. Classical Inflammatory Cytokines

Inflammatory processes are widely recognized as a critical contributor to cognitive impairment. C-reactive protein (CRP), which derives its name from its capacity to precipitate the somatic C-polysaccharide of Streptococcus pneumoniae [51], was an acute-phase inflammatory marker that sensitively reflected systemic inflammatory status [52]. Elevated CRP levels have consistently been observed in patients with cognitive impairment and dementia (Table 1), with higher CRP concentrations being associated with poorer cognitive performance [53]. A cross-sectional observational study indicated that MMSE scores are negatively correlated with plasma C-reactive protein concentrations in stroke survivors [54]. Multivariate logistic regression analysis further demonstrated that lower hs-CRP levels were associated with better cognitive outcomes following cerebral infarction [55].

Interleukin-6 (IL-6) is a crucial inflammatory biomarker [75]. A study indicated that IL-6 is significantly associated with cognitive deterioration at one year after stroke in patients with acute ischemic stroke or transient ischemic attack (TIA). Individuals with the highest IL-6 levels exhibited a 95% greater risk of post-stroke cognitive decline compared with those at the lowest levels. Elevated IL-6 concentrations were linked to a higher likelihood of reduced Montreal Cognitive Assessment (MoCA) scores in patients with ischemic stroke or TIA [56,59]. Moreover, research has shown that longitudinal variations in plasma IL-6 following ischemic stroke independently predict long-term cognitive outcomes. Notably, a doubling of IL-6 levels between admission and 6–9 months was associated with an eightfold increase in the risk of global cognitive impairment at 18–21 months, as well as declines in memory function [57]. Furthermore, higher concentrations of IL-8, IL-18, IL-10, IL-12, IL-18, IL-1β, and MIP-1α are significantly associated with reduced MoCA scores. IL-8, IL-18, IL-10, IL-12, IL-18, IL-1β and MIP-1α were informative biomarkers of pathogenic types of PSCI [58,60,61].

Rheumatoid factor (RF) is an autoantibody directed against the Fc portion of IgG [76]. Elevated RF concentration has been linked to a higher risk of PSCI [62,63].

Serum amyloid A (SAA) is a highly conserved acute-phase protein, which plays a role in attracting inflammatory cells through chemotaxis. SAA concentrations rise sharply during infection, injury, or inflammatory conditions [64]. Higher SAA levels are strongly linked to a greater risk of PSCI at 3 months and patients with more severe PSCI exhibit higher SAA concentrations than those with cognitively normal ischemic stroke [64].

#### 4.1.2. Innate Immune Activation and Immunometabolism-Related Biomarkers

Translocator protein (TSPO) is an outer mitochondrial membrane transmembrane protein that was first characterized as the peripheral benzodiazepine receptor [77]. TSPO expression is regarded as an indicator of brain damage and neuroinflammatory activity in various neurological conditions, such as encephalitis, stroke, and head trauma [78]. Serum TSPO levels above 2.18 ng/mL were linked to a higher likelihood of cognitive impairment. In addition, TSPO concentrations, which showed a significant inverse relationship with MoCA scores, were significantly higher in patients with cognitive impairment at 3 months following intracerebral hemorrhage (ICH) [65].

High mobility group box 1 (HMGB1) is a ubiquitous protein present in both the nucleus and cytoplasm compartments of nearly all cell types, where it plays an essential role in regulating gene expression and maintaining chromatin structure under homeostatic conditions. HMGB1 has been identified as an important contributor to deleterious inflammatory processes in acute brain injuries such as stroke and is also associated with the pathogenesis of several neurodegenerative disorders [79]. Inhibition of HMGB1 has been shown to improve learning and memory outcomes following stroke in a rodent model [80]. Some studies have suggested that higher plasma HMGB1 concentrations are linked to a greater likelihood of post-stroke cognitive deterioration in humans [81]. Findings from a prospective cohort investigation demonstrated that serum HMGB1 concentrations assessed within 24 h of acute ischemic stroke onset were linked to a higher likelihood of cognitive impairment. In addition, early post-stroke elevations in HMGB1 served as an independent predictor of worse MoCA scores at the 3-month follow-up [66]. In animal models of ischemic brain injury, increased HMGB1 expression has been associated with the onset of cognitive impairment [67].

The receptor for advanced glycation end products (RAGE) is present on the cell membrane of multiple cell types [82]. Multiple ligands, such as advanced glycation end products (AGEs) and HMGB1, interact with RAGE. Importantly, a soluble form of RAGE (sRAGE) exists, which is generated through alternative splicing of RAGE messenger ribonucleic acid (RNA) to produce the endogenous soluble form (esRAGE) or via proteolytic cleavage of the full-length RAGE protein [83]. Research has shown that patients with ischemic stroke (IS) who develop cognitive impairment exhibit markedly elevated concentration of sRAGE and esRAGE relative to individuals without cognitive deficits [68].

The triggering receptor expressed on myeloid cells-1 (TREM-1) is an immune receptor primarily expressed on neutrophils and monocytes [84]. It plays a key role in amplifying innate immune responses by acting synergistically with toll-like receptors in both infectious and non-infectious conditions [85]. Experimental evidence suggests that the inhibition of TREM-1 can improve long-term hippocampal function by modulating cellular proliferation and synaptic plasticity [86]. Moreover, findings from a prospective cohort study revealed that higher serum sTREM-1 concentrations independently predicted cognitive impairment after acute ischemic stroke [69]. Triggering receptor expressed on myeloid cells 2 (TREM-2) is a single-pass transmembrane protein predominantly expressed in myeloid-lineage cells. Within the central nervous system, it is chiefly expressed in microglia cells and plays a regulatory role in key events, including cell survival, proliferation, phagocytosis, and inflammatory responses [87]. Evidence from experimental studies indicate that sTREM2 improves microglial viability and induces inflammatory cytokine release, especially in TREM2-deficient microglial cells or mouse models [88]. In addition, the likelihood of PSCI increases with rising plasma sTREM2 levels, highlighting its utility for early diagnosis and intervention [70].

Galectin-3 is a pleiotropic protein that belongs to the lectin family and can be combined with β-galactoside [89] and acts as an important regulator of critical processes in acute and chronic inflammatory conditions [90]. Galectin-3 takes part in multiple biological processes, including inflammation, fibrosis and apoptosis [91]. A study indicated that AIS individuals in the PSCI group exhibited higher galectin-3 levels than those in the non-PSCI group. In addition, PSCI incidence increased in parallel with galectin-3 concentrations, indicating that circulating galectin-3 may be linked to PSCI risk [71].

The NOD-, LRR- and pyrin-domain-containing protein 3 (NLRP3) inflammasome is an inflammatory protein; it facilitates the maturation and release of pro-inflammatory cytokines and can trigger pyroptotic cell death and is mainly found in astrocytes and microglia. The inhibition of NLRP3 at early stages may reduce inflammatory responses, stabilize the BBB, and limit ischemia–reperfusion damage [92]. Patients with PSCI exhibited markedly increased plasma NLRP3 levels relative to healthy individuals. In addition, elevated concentrations of these inflammatory proteins showed an inverse correlation with overall and domain-specific MoCA scores [93].

The neutrophil–lymphocyte ratio (NLR) is a readily accessible marker of systemic inflammation. A study found that acute-phase NLR in ischemic stroke patients independently predicted PSCI at 3 months. Individuals with the highest NLR exhibited both global cognitive decline and impairments in memory and visuospatial domains. Notably, NLR levels above 3.80 were significantly linked to PSCI occurrence, indicating that NLR ≥ 3.80 may reflect an excessive systemic inflammatory state in the stroke population [72]. A separate study found that patients with PSCI exhibited higher levels of NLR, globulin-to-lymphocyte ratios (GLR), and C-reactive protein-to-lymphocyte ratios (CLR), while the lymphocyte-to-monocyte ratio (LMR) was significantly lower than that observed in the post-stroke cognitively normal (PSNCI) group [73].

Acute ischemic stroke triggers a systemic inflammatory reaction accompanied by increased concentrations of various pro-inflammatory cytokines [94]. Pro-inflammatory cytokines can activate the indoleamine-2,3-dioxygenase (IDO) enzyme, which leads to the depletion of tryptophan (TRP) levels and enhanced kynurenine formation, consequently increasing the peripheral blood kynurenine/tryptophan (K/T) ratio as a measurable marker of IDO enzymatic activity [95]. Research indicates that higher K/T ratios are associated with greater cognitive impairment in individuals with acute ischemic stroke [74].

Inflammatory biomarkers are particularly relevant during the acute and early sub-acute phases after stroke, when systemic immune activation and neuroinflammatory responses are most prominent. Therefore, markers such as CRP, IL-6, TNF-α, and peripheral inflammatory ratios may primarily serve as early predictors of subsequent cognitive decline by reflecting the initial inflammatory burden.

### 4.2. Oxidative Stress and Metabolic Biomarkers

#### 4.2.1. Oxidative Stress-Related Biomarkers

Superoxide dismutase (SOD) is a group of oxidoreductase enzymes that catalyze the conversion of superoxide radicals into hydrogen peroxide (H_2_O_2_) and oxygen (O_2_), playing an essential role in antioxidant defense and anti-inflammatory processes [96]. Decreased SOD may worsen brain injury following stroke and thus play a role in cognitive decline. At the same time, reduced SOD impairs antioxidants and anti-inflammatory defenses, while post-stroke inflammatory activation further contributes to secondary cerebral injury. A study indicated that patients with cognitive impairment showed markedly reduced serum SOD concentrations in the acute stage and at 3 months following mild AIS relative to those with normal cognition, accompanied by increased systemic inflammatory markers (Table 2). In addition, cognitive performance showed a positive correlation with serum SOD levels [97].

A prospective study indicated that patients with PSCI exhibited significantly lower total iron-binding capacity (TIBC) levels. In addition, elevated serum TIBC was linked to improved overall cognition, episodic memory, and language function [98].

8-hydroxydeoxyquanosine (8-OHdG) and malondialdehyde (MDA) are widely recognized as end products of deoxyribonucleic acid (DNA) oxidation and lipid peroxidation, which are measurable in human body fluids and brain [117]. Acute ischemic stroke is accompanied by a systemic oxidative stress response characterized by elevated levels of 8-OHdG and MDA. Several studies have indicated a link between oxidative stress and cognition [118]. Elevated levels of 8-OHdG and MDA are widely regarded as indicators of oxidative stress activation. Oxidative stress may contribute to PSCI through different biological mechanisms. A study found that serum levels of both 8-OHdG and MDA were markedly elevated in PSCI patients compared to those without PSCI, and both markers showed inverse correlations with MMSE scores. 8-OHdG and MDA exhibited considerable diagnostic value in differentiating PSCI from non-PSCI [99].

#### 4.2.2. Lipid and Energy Metabolism Biomarkers

Polyamines, such as putrescine, spermidine, and spermine, have recently attracted considerable attention and are thought to play essential roles in autophagy, cellular homeostasis, and cognitive function [119]. A study reported that higher baseline plasma levels of putrescine, spermidine, and spermine were linked to a greater risk of developing PSCI, showing a linear dose–response relationships [100]. Ischemic stroke patients with elevated polyamine levels should be continuously monitored and considered for early interventions to reduce the risk of PSCI.

Gamma-glutamyl transferase (GGT) serves as a serum metabolic biomarker and acts as an indicator of the body’s oxidative–antioxidant status that is involved in maintaining physiological concentrations of intracellular glutathione homeostasis and safeguarding cells against oxidative stress [120,121]. GGT warrants attention in studies related to PSCI. A large prospective cohort study demonstrated an inverse association between baseline GGT levels and the risk of PSCI. Notably, the association between GGT and PSCI requires careful interpretation. Markedly low GGT concentrations were identified as a risk factor for PSCI; this paradox may reflect the complex role of GGT in maintaining glutathione metabolism and antioxidant capacity [101].

Fibroblast growth factor 21 (FGF-21), belonging to the endocrine fibroblast growth factor subfamily, is an emerging metabolic regulator predominantly expressed and secreted by the liver and secondarily in the adipocytes [122]. Moreover, evidence suggests that plasma FGF-21 levels are higher in individuals with ischemic stroke. Elevated FGF-21 has been correlated with PSCI at 3 months, as well as with increased risks of mortality and profound disability following acute IS [102,103].

Serum albumin is a major protein in the blood that plays an important role in maintaining nutrition [123]. Studies indicated that it can improve cerebral circulation and provide protective effects on neurons and glial cells [124]. Lower serum albumin levels have also been linked to worse functional outcomes after stroke [125]. A meta-analysis found that individuals with PSCI had markedly reduced albumin levels compared with PSNCI patients [7].

The triglyceride/high-density lipoprotein cholesterol (TG/HDL-C) ratio was thought to significantly associate with insulin resistance [126], since it gained broad recognition as an important risk factor for cardiovascular events [127]. Elevated TG/HDL-C ratio was independently linked to an elevated likelihood of PSCI. The optimal cutoff value for predicting PSCI was 1.564 [104]. The triglyceride–glucose (TyG) index was also considered a reliable surrogate biomarker of insulin resistance (IR) [128]. Elevated TyG index values at admission were independently bound with an elevated risk of PSCI after 3 months and may serve as a potential predictive indicator [105].

Dipeptidyl peptidase-4 (DPP4) is a serine protease expressed in cell membranes and also in a soluble form (sDPP4) in plasma [129]. DPP4 functionally cleaves dipeptides from N terminus of peptides and has been proposed to inactivate intestinal hormones and consequently modulate glucose metabolism, cellular migration and differentiation, signal transduction, and immune function [130]. An earlier experimental study showed the inhibition of DPP4 with linagliptin-ameliorated transient-cerebral-ischemia-induced brain atrophy and cognitive deficits in diabetic mice [131]. A prospective multicenter study demonstrated that lower plasma sDPP4 levels were associated with a higher risk of PSCI in non-cardioembolic stroke patients, even after controlling for several known confounding factors [106].

L-carnitine has demonstrated neuroprotective properties in cerebral ischemia, primarily through improving mitochondrial function and attenuating inflammation. Supplementation with L-carnitine has also been suggested to enhance cognitive performance. Lower plasma L-carnitine levels were linked to an elevated risk of cognitive impairment for 3 months following ischemic stroke. Moreover, a combined effect between L-carnitine and inflammatory markers has been reported, where individuals with elevated L-carnitine and reduced inflammation presented the lowest risk of post-stroke cognitive impairment [107].

#### 4.2.3. Amino Acid Metabolism and Metabolic Regulatory Biomarkers

Homocysteine (Hcy) is a non-proteinogenic sulfhydryl-containing amino acid derived from methionine and is a homolog of cysteine [132]. It has been identified as a separate risk factor for dementia among the general public [133]. Individuals with Hcy concentrations exceeding 15 μmol/L had a 2.8-fold increased risk of cognitive decline compared with those with levels under 10 μmol/L [134]. Several studies have identified a meaningful link between Hcy and PSCI. Patients with PSCI tend to exhibit higher serum Hcy levels, and elevated Hcy has also been associated with an increased risk of post-stroke cognitive impairment [62,108,109].

Uric acid (UA) is the end catabolite of purine metabolism and exhibits multiple antioxidant properties [135]. Elevated oxidative stress is associated with increased serum UA levels and may shift the antioxidant properties of UA toward a pro-oxidative effect [136,137]; increased oxidative stress activity may contribute to cognitive impairment [138]. A study demonstrated that serum UA and Hcy levels were markedly elevated in PSCI patients compared with non-PSCI patients [110]. A cutoff value of 363.58 μmol/L for serum UA demonstrated high sensitivity and specificity for predicting PSCI. This indicates that serum UA could be a reliable predictor of cognitive impairment in patients with minor ischemic stroke [111]. Furthermore, a lower SUA/SCr ratio was linked to a higher prevalence of PSCI, suggesting that individuals with reduced SUA/SCr levels were at greater risk of post-stroke cognitive impairment [112].

Trimethylamine-N oxide (TMAO), a metabolite derived from dietary precursors via gut microbiota metabolism, has been implicated in vascular diseases [113]. Higher concentrations of TMAO have been shown to predict an increased risk of cardiovascular events [139] also implicated in ischemic brain injury [140]. A study demonstrated significant differences in MMSE scores across increasing quartiles of TMAO concentration, suggesting that patients with acute ischemic stroke and higher TMAO concentrations had an increased likelihood of developing PSCI [113].

N-acetylneuraminic acid (Neu5Ac) activates microglia-driven neuroinflammation and oxidative stress. Following cortical stroke, Neu5Ac accumulates in the hippocampus, where it exacerbates microglial oxidative stress and inflammation, thereby contributing to hippocampal secondary neurodegeneration and long-lasting cognitive impairment [114].

The PAr index can serve as a biomarker of inflammation and is positively associated with CRP, neopterin, and white blood cell counts [141]. Elevated baseline levels of the neuroexcitatory KP metabolites 3-hydroxykynurenine (HK) and quinolinic acid (QA) were significantly linked to poorer MoCA scores. Following the exclusion of individuals with pre-existing cognitive impairment, infection, or increased CRP at Day 1, higher baseline HKr remained significantly associated with MoCA scores at 36 months. QA was identified as a metabolite significantly linked to PSCI, though this association was observed only in the acute phase. Another neuroprotective KP metabolite picolinic acid (Pic), evaluated at 3 months post-stroke, was correlated with a more favorable cognitive status [115]. Serum quinolinic acid (QUIN) levels and the QUIN/KYNA (kynurenic acid) ratio were elevated and positively correlated with impaired cognitive performance on a spatial memory task. In addition, a trend towards a correlation between increased QA/KA ratios and decreased MoCA scores at 3 and 18 months were observed. Overall, serum QUIN concentrations and the QUIN/KYNA ratio appear to be reliable biomarkers for predicting PSCI [116].

Oxidative stress-related biomarkers may reflect both acute ischemic injury and persistent biological alterations during the post-stroke recovery phase. Early after stroke, increased oxidative damage markers may indicate acute cellular stress and mitochondrial dysfunction, whereas persistent abnormalities may reflect ongoing redox imbalance associated with neurodegeneration and impaired neural repair. Therefore, these biomarkers may have potential value for early risk prediction and the monitoring of biological recovery processes.

### 4.3. Vascular Injury and Endothelial Dysfunction Biomarkers

#### 4.3.1. Blood–Brain Barrier Disruption-Related Biomarkers

The matrix metalloproteinase (MMP) family of zymogen proteases, which is typically involved in extracellular matrix degradation and remodeling, may also contribute to the pathogenesis of various central nervous system disorders [142]. Matrix metalloproteinase-9 (MMP-9) is a central determinant of extracellular matrix degradation and is the most extensively studied member of the MMP family in acute IS. Both pro- and active MMP-9 levels are elevated within hours to days following focal stroke in humans, nonhuman primates, rats, and mice; these changes are temporally and spatially associated with the disruption of BBB integrity. Moreover, post-ischemic BBB disruption is attenuated by the inhibition of MMP activity or knockout of the MMP-9 gene [143]. A growing body of findings indicated that increased MMP-9 levels were involved in key pathological mechanisms, including inflammatory responses, BBB damage, and neuronal death, thereby potentially leading to cognitive dysfunction [144]. A study reported that patients with elevated MMP-9 levels had a significantly increased risk of cognitive impairment after stroke and increased serum MMP-9 concentrations in the early stage (Table 3) were significantly correlated with subsequent cognitive decline [145].

Tissue inhibitor of metalloproteinase-1 (TIMP-1) is a powerful endogenous inhibitor of MMP-9 and participates in a range of pathological processes [160]. TIMP-1 is produced by neurons, microglia, astrocytes, or endothelial cells, and cooperates with MMP-9 in modulating extracellular matrix remodeling within the central nervous system [161]. Elevated TIMP-1 and MMP-9 concentrations may damage the microvascular basal layer and contribute to leukoaraiosis, which can subsequently lead to cognitive dysfunction [162]. Elevated serum TIMP-1 concentrations were linked to a greater likelihood of cognitive impairment at 3 months following acute IS [146].

Cognitive outcomes were assessed with the Telephone Interview for Cognitive Status—modified (TICS-m) between 1 and 3 years after stroke. Adjusted linear mixed model analyses showed that higher log-transformed vascular cell adhesion molecule (VCAM-1) concentrations were associated with lower TICS-m scores over time. Consistently, among patients with mild-to-moderate first-ever ischemic stroke, increased baseline VCAM-1 levels were related to worse cognitive performance throughout the 3-year follow-up [147].

#### 4.3.2. Angiogenesis and Vascular Remodeling Biomarkers

Cystatin C (CysC) is an endogenous cysteine protease inhibitor that is continuously generated by all nucleated cells [163]. CysC has been implicated in the development of atherosclerosis and the destabilization of atherosclerotic plaques [164]. Reduced CysC level together with elevated cysteines protease and amyloid protein aggregation has direct cytotoxic effects on the brain and leads to cognitive decline. Conversely, elevated CysC levels have been shown to predict a higher risk of PSCI at 3 months of follow-up [148,149].

Vascular endothelial growth factor (VEGF) is a dimeric glycoprotein with multiple effects, including angiogenic and neuroprotective effects [165]. VEGF is a biomarker that becomes detectable within 2–4 h following stroke onset and can persist for at least 28 days [166]. VEGF plays a central role in promoting angiogenesis after ischemia occurs [167]. VEGF levels peak on day 7 following stroke [168]. Among patients with acute IS, those with VEGF levels ≥ 519.8 pg/mL had a higher risk of PSCI at 3 months post-onset. Moreover, elevated VEGF concentrations in PSCI individuals were linked to dysfunctions in visuospatial and recall functions [150].

Endostatin is considered a potent endogenous inhibitor of angiogenesis and originates from collagen XVIII [169]. Evidence from a study indicated that higher plasma endostatin levels were linked to cognitive impairment 3 months after acute ischemic stroke. Elevated endostatin may contribute to ischemic stroke pathology, possibly through inhibition of angiogenesis, induction of endothelial cell apoptosis, and impairment of blood–brain barrier integrity [151].

Alkaline phosphatase (ALP) is a metalloenzyme encoded by a multigene family and is widely distributed in both prokaryotic organisms and higher eukaryotic cells [170]. ALP is a homologous dimeric enzyme. In mammals, it exists in four isoforms, namely germ-cell, placental, intestinal, and tissue non-specific types [171]. The ALP subtype associated with cognitive function is classified as tissue non-specific type. Patients with PSCI had significantly elevated serum ALP levels compared with non-PSCI patients, and a non-linear dose–response relationship was identified between ALP concentrations and cognitive impairment [152].

Osteoprotegerin (OPG) is a soluble glycoprotein belonging to the tumor necrosis factor receptor superfamily and plays a key role in bone metabolism [172]. OPG has been associated with multiple inflammatory diseases and is expressed across different tissues, such as bone, vascular tissue, and immune cells [173]. Elevated plasma OPG levels were associated with an increased risk of PSCI at 3 months, suggesting that OPG may have served as a useful biomarker for predicting PSCI [153].

#### 4.3.3. Hemodynamic and Coagulation-Related Biomarkers

Fibrinogen (FIB) is an essential coagulation factor and a non-active precursor of fibrin [174]. FIB is present at higher levels in stroke patients than in non-stroke individuals [175]. Another study demonstrated that fibrinogen regulates the TGF-β receptor pathway in astrocytes. Activated astrocytes triggered by FIB release TGF-β, which acts on neurons to inhibit neurite outgrowth, ultimately leading to cognitive impairment [176]. Elevated plasma FIB concentrations were linked to the occurrence and the severity of PSCI [154,155].

Hemoglobin (Hb) is the primary protein responsible for transporting oxygen to tissues across the entire body, and the brain consumes about 20% of the body’s oxygen supply. Reduced hemoglobin levels cause cerebral hypoxia, which leads to diminished metabolic and neuronal activity in the brain tissue, subsequently contributing to mitochondrial dysfunction, oxidative stress, and inflammatory responses and, eventually, cognitive impairment [177]. Reduced Hb levels at admission were independently linked to a higher likelihood of PSCI and worse MMSE performance following stroke, with PSCI patients showing significantly lower Hb concentrations than those without cognitive impairment. Additionally, it has been proposed that keeping Hb levels above 15.0 g/dL in men during the acute stage of stroke may be favorable in lowering PSCI risk [156,157,158].

Thrombomodulin is a transmembrane protein constitutively expressed on endothelial cells and has been demonstrated to exert a variety of favorable biological effects on the vasculature [178]. Thrombomodulin is essential for preserving intravascular patency owing to its anticoagulant, anti-inflammatory, and cytoprotective properties [179]. Circulating thrombomodulin is able to interact with various proteins, thereby preventing coagulation and reducing inflammation, and therapeutics based on soluble thrombomodulin have been utilized for the treatment of neurological disorders associated with BBB dysfunction [180]. A study reported an inverse association between plasma thrombomodulin levels and the risk of PSCI at 3 months, along with a clear linear dose–response relationship [159].

The clinical relevance of vascular-related biomarkers is highly dependent on the timing after stroke. Biomarkers associated with endothelial dysfunction and BBB disruption and adhesion molecules may be most informative during the acute and sub-acute phases when vascular injury and barrier dysfunction are prominent. In later stages, persistent vascular abnormalities may contribute to chronic cognitive decline.

### 4.4. Neuronal Injury, Neurodegeneration and Synaptic Dysfunction Biomarkers

#### 4.4.1. Markers of Neuronal Structural Injury

Neurofilaments (Nfs) are key proteins that are synthesized in the somata of neurons and subsequently assembled into axonal intermediate filaments. They are essential for sustaining the morphology and stability of axons. Neurofilaments consist of three main forms: the neurofilament light chain (NfL), medium chain (NfM), and heavy chain (NfH) [181]. The concentration of NfL in cerebrospinal fluid or peripheral blood is considered a biomarker of axonal injury and neurodegeneration in various neurological disorders [182]. Neurofilament light chain (NfL) is a neuron-specific structural protein [183] that has recently been proposed as a biomarker of axonal injury and it may be applied potentially in patient monitoring as well as both observational and interventional research [184]. Individuals with PSCI had elevated phosphorylated NfL (pNfL) and Hcy levels that were higher than those without PSCI (Table 4). Moreover, pNfL was inversely associated with MoCA and showed strong diagnostic accuracy in differentiating PSCI from non-PSCI [12,185]. The phosphorylation of NfH regulates axonal diameter and safeguards neurofilament proteins against degradation. Impaired NfH phosphorylation in neurons may disrupt axonal transport and ultimately lead to neuronal cell death. Phosphorylated NfH (pNfH) has been identified as a dependable serum biomarker of axonal damage [186]. In the acute stage, serum pNfH concentrations were markedly increased in stroke individuals compared with healthy individuals and showed associations with stroke lesion volume and NIHSS scores [187].

Tau protein is a microtubule-associated protein that is widely expressed in the central nervous system, where it plays a key role in maintaining neuronal structure and regulating neuronal function. The pathological aggregation and accumulation of tau protein represent features of multiple neurodegenerative disorders [199]. Tau levels at 3 months were lower in individuals who developed PSCI at 1 year compared with those without PSCI [188]. Elevated plasma p-tau181 levels during the acute post-stroke stage were linked to a reduced risk of PSCI at 3 and 12 months [188]. Cognitive impairment after stroke may result from vascular cognitive impairment caused by cerebrovascular injury, pre-existing Alzheimer’s disease-related pathology, acute neuronal damage induced by stroke, impaired clearance of pathological proteins due to BBB dysfunction, or a combination of these processes. Therefore, changes in circulating Aβ and tau after stroke may reflect both stroke-related secondary neuronal injury and underlying neurodegenerative vulnerabilities that existed before stroke.

The underlying mechanism of Aβ1-42 level for PSCI has been investigated in several studies. Experimental findings indicate that acute ischemic injury increases amyloid precursor protein expression and Aβ generation in the early phase after stroke, leading to facilitating Aβ deposition and clearance in the brain [200]. Post-stroke impairment of perivascular space integrity, along with inflammation, hypoxia, and BBB disruption, may accelerate Aβ deposition in both brain parenchyma and cerebral vessel walls or worsen cerebral amyloid angiopathy (CAA). Parenchymal Aβ accumulation is thought to act as a triggering event, contributing to impaired synaptic function and ultimately contributing to cognitive decline and dementia [201]. Patients with PSCI exhibited markedly reduced blood Aβ1-42 levels compared with non-PSCI individuals, suggesting that Aβ1-42 was inversely correlated with PSCI [190,202,203]. Following stroke onset, serum Aβ1-42 levels are markedly decreased owing to the precipitation effect. Moreover, prior research has indicated that the plasma Aβ42/40 ratio can act as a surrogate biomarker of cortical Aβ deposition [204]. Patients with PSCI exhibited lower plasma Aβ1-42 concentration at 3 months, which was a powerful predictor of PSCI [188,191].

Glial fibrillary acidic protein (GFAP) is a brain-specific intermediate filament protein predominantly expressed in astrocytes and is secreted from damaged or activated astrocytes [205]. GFAP is essential for neuron protection, BBB maintenance, and reactive gliosis [206]. An increasing body of evidence suggests the promising clinical utility of GFAP concentrations in a range of neuroinflammatory and neurodegenerative diseases [207]. In patients with ischemic stroke, GFAP levels showed an independent positive association with PSCI, and elevated GFAP was also associated with a higher risk of cognitive impairment at 90-day follow-up [93,192].

Neuroglobin is a monomeric hexametric heme protein, belonging to the globin family and predominantly expressed in neurons of the central and peripheral nervous systems [208]. As a recently identified neuroprotective protein, neuroglobin has shown promising clinical applications. Research has shown that serum neuroglobin concentrations are markedly reduced in PSCI patients compared with non-PSCI individuals after intracranial hemorrhage (ICH). In addition, neuroglobin exhibits strong sensitivity and specificity in predicting PSCI after ICH, indicating its usefulness as a predictive biomarker [193].

#### 4.4.2. Synaptic Dysfunction and Neuroplasticity-Related Biomarkers

Brain-derived neurotrophic factor (BDNF), a key molecule of neurotrophins family in the central nervous system, is essential for the differentiation and survival of neuronal cells [209]. BDNF plays a central role in maintaining synaptic plasticity and promoting synaptogenesis, processes that underlie the cellular mechanisms of acquisition and stabilization of memory [210]. Lower BDNF concentrations can contribute to neuronal degeneration, which may result in hippocampal atrophy and subsequent cognitive impairment [211]. IS patients in the highest tertile of BDNF exhibited a 40% reduction in the likelihood of 3-month PSCI in comparison with those in the lowest tertile [194].

Neuronal pentraxin 2 (NPTX2) belongs to the pentraxins family, which comprises a signal peptide sequence and three N-glycosylation sites [212]. NPTX2 associates with neuronal pentraxin 1 (NPTX1) and the neuronal pentraxin receptor (NPTXR) to constitute multimeric complexes that facilitate synaptogenesis and synaptic remodeling in cortical regions and hippocampal pyramidal neurons [213]. NPTX2 plays a crucial role in synapse formation, synaptic remodeling, and excitatory synaptic transmission [214]. NPTX2 has emerged as an innovative biomarker of synaptic impairment linked to cognitive function in neurodegenerative diseases. A study reported that reduced NPTX2 concentrations were markedly associated with PSCI, particularly with deficits in visuospatial and executive domains. Additionally, a serum NPTX2 level below 0.986 ng/mL was identified as the ideal threshold for supporting the diagnosis of PSCI [195].

Retinoic acid (RA) is a major metabolite of vitamin A, including carotenoids and retinyl esters, plays an essential role in growth and development. Its diverse biologic effects are mediated through binding to nuclear receptors, such as retinoic acid receptors and the retinoid X receptor, thereby regulating specific gene cassettes [215,216]. In addition, the administration of RA has been shown to promote functional recovery and endogenous repair, which may facilitate increased synaptic plasticity after ischemic stroke. Reduced RA concentrations have been suggested as an independent predictor of poor long-term prognosis following ischemic stroke [217]. Lower baseline serum RA levels were independently linked to an elevated risk of PSCI at 3 months following ischemic stroke [196].

There were higher serum β-secretase enzyme (BACE1) levels and lower soluble receptor for advanced glycation end products (sRAGE) levels in individuals with post-stroke cognitive dysfunction than in patients with stroke only. These levels were altered at stroke onset, whereas cognitive dysfunction could be clinically diagnosed two weeks post-stroke, indicating that BACE1 and sRAGE served as useful biomarkers for predicting post-stroke cognitive impairment and facilitating early treatment [197].

#### 4.4.3. Neuroregulatory and Neurotransmitter-Related Biomarkers

A decline in thyroid hormone levels can adversely affect cerebral blood flow perfusion and energy metabolism, which may result in widespread neurological dysfunction and cognitive impairment [218]. Repeated-measures ANOVA showed that triiodothyronine (T3) concentrations were lower in patients with PSCI compared with those without PSCI. These findings suggest that post-stroke changes in T3 may be linked to disease progression and may serve as a biomarker for long-term dynamic monitoring and risk evaluation [191].

Neuropeptide Y (NPY) is a 36-amino acid peptide neurotransmitter that is produced in large quantities and widely expressed in the mammalian nervous system [219]. Furthermore, NPY has also been indicated to act as a neuromodulator of cognitive performance, exhibiting particular memory-improving effects and the ability to ameliorate scopolamine-induced amnesia [220]. Furthermore, experimental evidence indicates that intracerebroventricular injections of NPY fragments and analogs promote learning and memory related cognitive function, and that a blockade of NPY receptors can abolish memory impairments [221]. NPY has been shown to modulate a variety of biological and pathophysiological processes, including neuroplasticity and neurotrophic support [222]. A study found that elevated NPY was correlated with a lower risk of post-acute IS cognitive impairment, with each one-standard-deviation increase in log-NPY corresponding to a 20% reduction in risk [198].

Compared with inflammatory and vascular biomarkers, neurobiological biomarkers may reflect relatively downstream consequences of stroke-related brain damage. Acute elevation in NfL, GFAP, and related markers may indicate axonal injury, astroglial activation, or tissue damage severity, whereas persistent elevation during follow-up may be associated with ongoing neurodegenerative processes and long-term cognitive decline. Therefore, these biomarkers appear particularly promising for prognostic prediction and the identification of individuals at high risk of persistent cognitive impairment.

### 4.5. Regulatory Molecules and Emerging Biomarkers

#### 4.5.1. Circulating Non-Coding RNAs

Exosomes are small membrane vesicles present in extracellular fluid and contain important genetic materials including DNA, RNA, protein, lipid and miRNA. Exosomes exist in extracellular fluids and contain important genetic materials such as DNA, RNA, protein, lipid and microRNA (miRNA) [223]. Research on non-coding RNAs (ncRNAs) has progressed speedily over the past few years—driven particularly by advances in high-throughput sequencing, whole-gene sequencing, and bioinformatics—most notably, miRNAs, small RNA molecules of 18–21 nucleotides that modulate downstream gene expression by cleaving target mRNAs or inhibiting their post-transcriptional translation.

MicroRNAs regulate a wide range of intra- and extracellular signal transduction pathways in the neocortex, hippocampus, and limbic systems. Specifically, miRNAs participate in the regulation of close junctions within the BBB and influence neuronal growth, proliferation, and differentiation. Moreover, miRNA expression profiles have been shown to be largely consistent among individuals within the same species [224]. miRNAs exist in human plasma in a highly stable form that protects them from endogenous RNase activity miRNA [225]. In comparison with positron emission tomography (PET) or the protein analysis of cerebrospinal fluid, miRNAs have the primary benefit of a non-invasive nature and are highly stable while providing large amounts of data [226]. There is accumulating evidence that miRNAs are critically involved in vascular, metabolic diseases and cancer and may serve as novel biomarkers and therapeutic targets across a range of pathological conditions [227]. Dysregulated miRNA expression in PSCI and related conditions can disrupt downstream gene networks, resulting in neuronal dysfunction and cognitive impairment [228]. A study reported that patients with acute stroke exhibited significantly lower MoCA scores compared with the normal group. Additionally, serum miR-195 and miR-497 levels were inversely associated with MoCA, indicating their potential as biomarkers of cognitive dysfunction in acute stroke patients [229]. Animal experiments demonstrated that the intravenous injection of miR-20a-3p enhanced recovery after acute stroke (Table 5) in both male and female rats and additionally mitigated post-stroke cognitive impairment in female rats [230]. A study demonstrated that miR-21, miR-132, and miR-200b were substantial diagnostic biomarkers of PSCI. Furthermore, miR-21 levels were positively associated with MMSE performance. MiR-132 is recognized as an important modulator of cognitive ability, playing a key role in memory formation and maintenance. MiR-200b has been linked to age-associated cognitive decline and stroke consequences in humans [231]. MiR-9, miR-29b, miR-124, and miR-125b-5p as forecasting biomarkers of cognitive recovery after ischemic stroke [232]. MiR-511–3p has been recognized as an independently predictive factor of PSCI, and shows a positive association with MoCA performance [233]. Clinical evidence indicates that significantly decreased miR-93 levels during the acute phase are independently correlated with cognitive impairment. Individuals with lower miR-93 expression tend to have poorer MoCA scores [234]. Recent studies found that miR-let-7i, miR-93-3p, miR-502-3p were significantly upregulated in PSCI patients and may function as reliable diagnostic biomarkers for PSCI [235,236,237]. In summary, a range of miRNAs including miR-21, miR-91, miR-93, miR-124, miR-132, miR-195, miR-497, miR-200b, miR-29b, miR-125b-5p, miR-511–3p, miR-216, miR-93-3p, Let-7f-5p, miR-let-7i, miR-502-3p and miR-223–3p have been identified as potential diagnostic or prognostic biomarkers for PSCI. Plasma exosome-derived miRNAs are emerging as promising tools for PSCI diagnosis [228].

Long non-coding RNAs (lncRNAs), a major component of the ncRNA family, have received considerable attention in recent epigenetics research. Prior research has reported abnormal lncRNA expression in neurodegenerative diseases and demonstrated lncRNAs are dysregulated in PSCI [242]. Serum SIX3OS1 levels in PSCI patients were negatively related to MoCA. Hence, elevated serum SIX3OS1 may represent a biomarker for PSCI development in acute stroke and is linked to more pronounced cognitive impairment [238]. NEXN-AS1 expression was markedly decreased in stroke patients and showed an even greater reduction in those with PSCI. It has been proposed as a reliable biomarker for differentiating stroke patients from healthy individuals and for distinguishing PSCI from post-stroke cognitive normality (PSCN) [239].

Circular RNAs (circRNAs), a newly identified subtype of non-coding RNAs, are abundantly expressed in the nervous system and are involved in the pathogenesis of IS through the regulation of neurogenesis, inflammatory responses, apoptosis, and atherosclerotic processes [243]. Owing to their remarkable stability, conservation, tissue organ specificity, developmental stage specificity, and high abundance in body fluids, they represent promising biomarkers and promising therapeutic targets for PSCI [244]. A study characterized circRNAs expression profiles in PSCI and suggested that hsa_circ_0089762, hsa_circ_0064644, and hsa_circ_0089763 could act as promising biomarkers for the early detection of PSCI in patients with large-artery atherosclerosis (LAA) anterior circulation infarction [240].

#### 4.5.2. Multi-Omics Biomarkers

Metabolomics is a molecular regulatory network and top-down platform that concentrates on the dynamic changes in endogenous metabolites as a response to biological perturbations. It stands as an effective strategy for capturing disease particular metabolic signatures that could be used as candidate biomarkers [245]. Metabolomics, through untargeted liquid chromatography–mass spectrometry (LC-MS) or nuclear magnetic resonance (NMR) platforms that provide high-resolution profiling of low-molecular-weight metabolites enable the discovery of biomarkers beyond the scope of traditional assays [246]. The combination of glutamine, kynurenine, and LysoPC (18:2) showed a strong ability to distinguish stroke patients with cognitive impairment from those without cognitive deficits, with good diagnostic accuracy. They are primarily involved in stroke-induced inflammation and neurotoxicity [245]. A systematic review revealed that six metabolic pathways were significantly enriched in relation to PSCI, including the biosynthesis of phenylalanine–tyrosine–tryptophan, the biosynthesis and degradation of valine–leucine–isoleucine, phenylalanine metabolism, tryptophan metabolism and nitrogen metabolism [247].

Microbiome analyses in PSCI have revealed specific gut microbial taxa with potential as non-invasive diagnostic biomarkers, such as Firmicutes, Bacteroidetes, Enterobacteriaceae, and Bacteroides. PSCI transcriptomic profiling has further underscored the diagnostic potential of several miRNAs, such as miR-21, miR-132, miR-195, miR-200b, miR-497, and miR-let-7i. Moreover, proteomic analyses have identified neuroglobin as a potential diagnostic biomarker for PSCI [248]. Histone deacetylase 4 (HDAC4) regulates gene transcription by modulating histone acetylation [249]. HDAC4 has also been implicated in the modulation of memory and cognitive impairment. For instance, a previous study reported that either the overexpression or knockdown of HDAC4 in the mushroom body, a critical brain structure for memory formation, disrupted long-term memory in drosophila. This indicates that normal HDAC4 expression is required for the preservation of long-term memory [250]. A separate study found that high HDAC4 expression in the hippocampus promoted neuronal apoptosis, which may have led to cognitive impairment [251]. Low HDAC4 levels are indicative of an increased risk of PSCI [241].

The temporal interpretation of non-coding RNA and multi-omics biomarkers remains challenging because most studies have evaluated these signatures at single time points and with heterogeneous analytical platforms. Emerging evidence suggests that these molecular signatures may capture early regulatory changes preceding overt cognitive decline and may therefore have the potential for early prediction and molecular stratification.

### 4.6. Holistic Understanding and Clinical Implications of Blood Biomarkers in PSCI

In recent years, with the continuous deepening of research on PSCI, many blood-based biomarkers have been successively identified and confirmed to be associated with cognitive decline. However, a review of existing studies reveals that although these biomarkers originate from different sources and have diverse functions, they essentially reflect a series of consecutive and interwoven pathophysiological processes triggered by post-stroke brain damage. Therefore, existing biomarkers are not independent of each other but reflect pathological changes at different stages and levels of the PSCI. Despite the increasing number of reported biomarkers, the interpretation and comparison of current evidence remain challenging due to substantial heterogeneity among existing studies. Differences in patient populations, stroke characteristics, blood sampling time points, analytical platforms, and cognitive assessment tools may contribute to inconsistent findings across cohorts. For example, biomarkers measured during the acute phase after stroke may primarily reflect initial tissue injury and systemic responses, whereas biomarkers assessed during the chronic phase may better represent neurodegenerative processes and long-term cognitive deterioration. Therefore, biomarker levels should be interpreted within the temporal and clinical context of stroke evolution rather than considered as universal indicators of PSCI.

Importantly, currently available biomarkers may serve different clinical purposes. Some inflammatory and vascular markers may be more suitable for early risk stratification after stroke. Notably, the most extensively studied markers are inflammation-related biomarkers, such as CRP, IL-6, TNF-α, and NLR, and have demonstrated strong associations with the risk of PSCI across multiple independent cohorts (Table 6). However, inflammation markers are generally plagued by insufficient specificity, as their levels are easily influenced by factors such as infection, underlying comorbidities, and stroke severity, thereby making them unsuitable as standalone diagnostic tools for PSCI. In contrast, neurobiological markers may provide additional information regarding ongoing brain damage and long-term cognitive decline. Neurobiological markers associated with neuronal damage and neurodegeneration, such as NfL, GFAP, Aβ, and Tau proteins, exhibit more direct biological associations with cognitive function. These markers can effectively reflect axonal injury, neuroglial activation, and neurodegenerative pathological changes, and thus are considered to have high potential for clinical translation. Meanwhile, distinguishing diagnostic biomarkers from prognostic biomarkers is essential for evaluating their clinical utility. Although neuronal injury biomarkers have shown promising associations with PSCI, their interpretation requires consideration of their relationship with overall stroke-related injury burden. NfL, pNfH, GFAP, and tau primarily reflect different aspects of neuronal and glial damage, including axonal injury, infarct burden, hemorrhagic injury, and BBB disruption. Therefore, elevated levels of these biomarkers may partly represent the severity of the initial stroke rather than cognitive-specific pathological processes. Importantly, whether these biomarkers provide additional predictive value beyond established clinical and imaging predictors, such as lesion volume, NIHSS score, stroke subtype, age, and education level, remains insufficiently clarified. Meanwhile, the emerging non-coding RNA and multi-omics research in recent years have brought new opportunities to PSCI research. Compared with traditional protein biomarkers, emerging technologies such as miRNA, lncRNA, circRNA, metabolomics, transcriptomics can reveal disease mechanisms at the level of molecular regulatory networks and are expected to identify early pathological changes that cannot be detected by conventional methods. However, most current studies are still in the exploratory phase, lacking reproducible validation of results and standardized detection methods, and thus remain a considerable distance from clinical application.

Blood-based biomarkers investigated in PSCI should be distinguished according to their clinical purpose. Biomarkers identified from studies comparing patients with established PSCI and those without cognitive impairment may have potential diagnostic value, including galectin-3, 8-OHdG, UA, ALP, Nfs, Aβ1-42, and neuroglobin, whereas biomarkers measured during the acute or early sub-acute phase after stroke are mainly evaluated for predicting subsequent cognitive decline. Notably, most currently available biomarkers, including inflammatory, vascular, and neuronal injury-related markers, show greater potential for risk prediction than for diagnosis of established PSCI, as their levels may also be influenced by stroke severity, systemic inflammation, and other comorbidities.

Given the multifactorial nature of PSCI, a single biomarker is unlikely to fully capture the complexity of post-stroke cognitive deterioration. Future approaches should move beyond individual biomarkers toward integrated models combining complementary biological domains, including inflammatory markers, vascular injury indicators, neuronal injury markers, metabolic signatures, and clinical characteristics. Integration with neuroimaging parameters, artificial intelligence algorithms, and multi-omics approaches may further improve risk prediction and patient stratification.

## 5. Current Issues and Future Directions

Early and accurate identification of PSCI is crucial for improving patients’ quality of life and clinical outcomes. Nevertheless, research efforts to develop effective blood-based biomarkers for PSCI have been hampered by several challenges. Most are observational in design, precluding causal inference and remaining susceptible to residual or unmeasured confounding despite multivariable adjustment. The predominance of single-center studies with small sample sizes, lack of independent external validation, and the exclusion of patients with severe conditions contribute to limited generalizability and potential selection bias. In addition, reliance on single time-point measurements fails to capture dynamic biomarker changes across the acute, sub-acute, and recovery phases, while relatively short follow-up durations constrain the evaluation of long-term cognitive outcomes and progression to dementia. Cognitive assessment is frequently based on a single screening tool, which lacks sensitivity for domain-specific and mild impairments and is influenced by educational and cultural factors. Moreover, most studies focus on individual biomarkers without integrating multimodal indicators, and mechanistic insights remain limited due to insufficient experimental validation, leaving uncertainty as to whether biomarker alterations are causal, consequential, or epiphenomenal. The moderate predictive performance of most biomarkers further limits their clinical utility when used alone.

Future research should prioritize large-scale, multicenter prospective cohorts with independent external validation to enhance robustness and generalizability. Longitudinal designs incorporating repeated biomarker measurements across multiple time points, alongside standardized and repeated cognitive assessments, are needed to elucidate temporal relationships with cognitive decline. Comprehensive neuropsychological batteries should be employed to improve detection of subtle and domain-specific deficits. In parallel, mechanistic studies integrating animal and cellular models are essential to clarify underlying molecular pathways. Lastly, the combined predictive value of different biomarkers should be evaluated and the efficacy differences between single biomarkers and combined models compared, combining imaging indicators such as brain MRI and PET [252] and integrating multi-omics data including genomics and metabolomics to improve the accuracy of prediction and diagnosis of PSCI.

Overall, current research on PSC blood biomarkers has gradually shifted from the stage of purely exploratory correlation studies to mechanistic research and clinical translation. The key future direction is not to discover more new biomarkers, but to screen core indicators with stability, reproducibility, and clinical applicability, and to establish comprehensive predictive models validated through standardized, multicenter approaches. This will enable the true realization of early identification, precise risk stratification, and individualized intervention for PSCI.

## 6. Conclusions

Research into blood-based biomarkers associated with PSCI is a rapidly evolving field, and it is expected that such biomarkers will play an important role in improving the clinical management of patients with PSCI in the future. A broad spectrum of potential biomarkers that reflect diverse pathophysiological aspects of the disease, including inflammation and immune, oxidative stress and metabolic dysregulation, vascular injury and blood–brain barrier disruption and neuronal injury/neurodegeneration and synaptic dysfunction. Among current candidates, IL-6, Hcy, MMP-9 and Aβ1-42 have shown relatively stronger evidence. Despite initial advances in the research on blood biomarkers for PSCI, current blood biomarkers remain insufficient for use as standalone diagnostic tools. Their clinical interpretation requires integration with established approaches, including cognitive assessment, neuroimaging findings, clinical history, stroke severity evaluation, and vascular risk factor assessment. Meanwhile, multiple methodological limitations and barriers to clinical translation still need to be overcome. Through integrated multicenter, longitudinal, multi-omics and interventional studies, it is expected to identify stable and reliable biomarkers, providing critical support for the early warning, precise intervention and prognosis evaluation of PSCI.

## Figures and Tables

**Table 1:** The inflammation and immune blood-based biomarkers of PSCI.

Mechanism	Biomarker	Expression Change	Sample Size	Blood Fraction	Cognitive Test	Method	Evidence Level	Ref.
Inflammation and Immune Biomarkers	CRP	Increase	48	Serum	MMSE	ELISA	Moderate	[54]
			197	Serum	MoCA	ELISA		[55]
	IL-6	Increase	238	Serum	MoCA	ELISA	High	[56]
			190	Plasma	MoCA	ELISA		[57]
			72	Serum	Unknown	ELISA		[58]
			455	Plasma	MoCA	ELISA		[59]
	IL-8	Increase	236	Plasma	MMSE	ELISA	Moderate	[60]
			243	Serum	Unknown	ELISA		[61]
	IL-10	Increase	72	Serum	Unknown	ELISA	Low	[58]
	IL-12	Increase	243	Serum	Unknown	ELISA	Low	[61]
	IL-18	Increase	236	Plasma	MMSE	ELISA	Low	[60]
	IL-1β	Increase	72	Serum	Unknown	ELISA	Low	[58]
	MIP-1α	Increase	455	Plasma	MoCA	ELISA	Low	[59]
	RF	Increase	638	Serum	MoCA	Immunological transmission turbidimetry	Moderate	[62]
			582	Serum	MoCA	Immunological transmission turbidimetry		[63]
	SAA	Increase	80	Serum	MoCA	ELISA	Low	[64]
	TSPO	Increase	276	Serum	MoCA	ELISA	Low	[65]
	HMGB1	Increase	192	Serum	MoCA	ELISA	Moderate	[66]
			16	Serum	Unknown	ELISA		[67]
	RAGE	Increase	172	Plasma	MoCA	ELISA	Low	[68]
	TREM-1	Increase	291	Serum	MoCA	ELISA	Low	[69]
	TREM-2	Increase	599	Plasma	MMSE	ELISA	Low	[70]
	Galectin-3	Increase	416	Serum	MoCA	ELISA	Low	[71]
	NLRP3	Increase	108	Plasma	MoCA	ELISA	Low	[71]
	NLR	Increase	345	Whole blood	MMSE	Complete blood count	Moderate	[72]
			146	Whole blood	MoCA	Complete blood count		[73]
	GLR	Increase	146	Whole blood	MoCA	Complete blood count	Low	[73]
	CLR	Increase	146	Whole blood	MoCA	Complete blood count	Low	[73]
	LMR	Decrease	146	Whole blood	MoCA	Complete blood count	Low	[73]
	K/T	Increase	41	Plasma	MMSE	HPLC–MS	Low	[74]

Abbreviations: CRP, C-reactive protein; IL, interleukin; MIP-α, macrophage inflammatory protein-1 alpha; RF, rheumatoid factor; SAA, serum amyloid A; TSPO, translocator protein; HMGB1, high mobility group box 1; RAGE, receptor for advanced glycation end products; TREM-1, triggering receptor expressed on myeloid cells-1; TREM-2, triggering receptor expressed on myeloid cells 2; NLRP3, NOD-, LRR- and pyrin-domain-containing protein 3; NLR, neutrophil–lymphocyte ratio; GLR, globulin-to-lymphocyte ratio; CLR, C-reactive protein-to-lymphocyte ratio; LMR, lymphocyte-to-monocyte ratio; K/T, kynurenine/tryptophan; ELISA, enzyme-linked immunosorbent assay; HPLC–MS, high-performance liquid chromatography–mass spectrometry. MMSE, mini-mental state examination; MoCA, Montreal cognitive assessment; evidence levels were based on the overall strength of available evidence, considering study number, cohort size, study design, replication, adjustment of confounding factors, diagnostic/prognostic performance, and clinical feasibility.

**Table 2:** The oxidative stress and metabolic blood-based biomarkers of PSCI.

Mechanism	Biomarker	Expression Change	Sample Size	Blood Fraction	Cognitive Test	Method	Evidence Level	Ref.
Oxidative Stress and Metabolic Biomarkers	SOD	Decrease	187	Serum	MoCA	Pyrogallol autoxidation	Low	[97]
	TIBC	Decrease	500	Serum	Unknown	Colorimetric method	Low	[98]
	8-OHdG	Increase	193	Serum	MMSE	ELISA	Low	[99]
	MDA	Increase	193	Serum	MMSE	Spectrophotometry	Low	[99]
	Polyamine	Increase	619	Plasma	MoCA and MMSE	UPLC-TQ-MS	Low	[100]
	GGT	Decrease	1957	Serum	MoCA	Unknown	Low	[101]
	FGF-21	Increase	3412	Plasma	Unknown	ELISA	Moderate	[102]
			600	Plasma	MoCA and MMSE	ELISA		[103]
	Serum albumin	Decrease	424	Serum	MoCA and MMSE	Unknown	Low	[7]
	TG/HDL-C	Increase	227	Plasma	MoCA	Unknown	Low	[104]
	TyG index	Increase	313	Plasma	MoCA	Unknown	Low	[105]
	sDPP4	Decrease	600	Plasma	MoCA	ELISA	Low	[106]
	L-carnitine	Decrease	617	Plasma	MoCA	UHPLC-MS/MS	Low	[107]
	Hcy	Increase	638	Plasma	MoCA	Enzymatic cycling assay	High	[62]
			325	Serum	MoCA	Unknown		[108]
			81	Serum	MMSE	ELISA		[109]
	UA	Increase	274	Serum	MoCA	Unknown	Moderate	[110]
			318	Serum	MoCA	Unknown		[111]
	SUA/SCr	Decrease	130	Serum	MoCA	Unknown	Low	[112]
	TMAO	Increase	256	Plasma	MMSE	ID-HPLC-MS/MS	Low	[113]
	Neu5Ac	Increase	148	Serum	Unknown	LC-MS/MS	Low	[114]
	PAr	Increase	422	Plasma	MoCA	LC-MS/MS	Low	[115]
	HKr	Increase	422	Plasma	MoCA	LC-MS/MS	Low	[115]
	HK	Increase	422	Plasma	MoCA	LC-MS/MS	Low	[115]
	QA	Increase	422	Plasma	MoCA	LC-MS/MS	Low	[115]
	QUIN	Increase	20	Serum	MoCA and MMSE	HPLC–MS	Low	[116]
	QUIN/KYNA ratio	Increase	20	Serum	MoCA and MMSE	HPLC–MS	Low	[116]

Abbreviations: SOD, superoxide dismutase; TIBC, total iron-binding capacity; 8-OHdG, 8-hydroxydeoxyquanosine; MDA, malondialdehyde; GGT, gamma-glutamyl transferase; FGF-21, fibroblast growth factor 21; TG/HDL-C, triglyceride/high-density lipoprotein cholesterol; TyG, triglyceride–glucose; sDPP4, soluble dipeptidyl peptidase-4; Hcy, homocysteine; UA, uric acid; SUA/SCr: serum uric acid/serum creatinine; TMAO, trimethylamine-N oxide; Neu5Ac, N-acetylneuraminic acid; PAr, an inflammatory marker based on vitamin B6 metabolites; HKr, a marker of functional vitamin B6 status based on selected KP metabolites; HK, 3-hydroxykynurenine; QA, quinolinic acid; QUIN, quinolinic acid; QUIN/KYNA, quinolinic acid/kynurenic acid. UPLC-TQ-MS, ultraperformance liquid chromatography coupled with triple quadrupole mass spectrometry; UHPLC-MS/MS, ultrahigh-performance liquid chromatography–tandem mass spectrometry; ID-HPLC-MS/MS, isotope dilution high-performance liquid chromatography with online tandem mass spectrometry; LC-MS/MS, liquid chromatography–tandem mass spectrometry.

**Table 3:** The vascular injury and endothelial dysfunction blood-based biomarkers of PSCI.

Mechanism	Biomarker	Expression Change	Sample Size	Blood Fraction	Cognitive Test	Method	Evidence Level	Ref.
Vascular Injury and Endothelial Dysfunction Biomarkers	MMP-9	Increase	236	Plasma	MMSE	ELISA	High	[60]
			638	Serum	MoCA	ELISA		[62]
			558	Serum	MoCA	ELISA		[145]
	TIMP-1	Increase	598	Serum	MoCA and MMSE	ELISA	Low	[146]
	VCAM-1	Increase	570	Serum	Unknown	Magnetic bead-based immunoassay	Low	[147]
	CysC	Increase	1025	Serum	MoCA	Immunoturbidimetric	Moderate	[148]
			281	Serum	MoCA	Immunoturbidimetric		[149]
	VEGF	Increase	56	Serum	MoCA	ELISA	Low	[150]
	Endostatin	Increase	613	Plasma	MoCA	ELISA	Low	[151]
	ALP	Increase	1019	Serum	MMSE	AMP	Low	[152]
	OPG	Increase	613	Plasma	MoCA and MMSE	ELISA	Low	[153]
	FIB	Increase	210	Plasma	MMSE	Coagulation assay	Moderate	[154]
			268	Plasma	BNIS	Automated clot rate assay		[155]
	Hb	Decrease	326	Whole blood	MMSE	Unknown	High	[156]
			1081	Whole blood	MMSE	Unknown		[157]
			2240	Whole blood	MoCA	Unknown		[158]
	Thrombomodulin	Decrease	615	Plasma	MoCA	ELISA	Low	[159]

Abbreviations: MMP-9, matrix metalloproteinase-9; TIMP-1, tissue inhibitor of metalloproteinase-1; VCAM-1, vascular cell adhesion molecule; VEGF, vascular endothelial growth factor; ALP, alkaline phosphatase; OPG, osteoprotegerin; FIB, fibrinogen; Hb, hemoglobin; AMP, 2-amino-2methyl-1-propanol; BNIS, Barrow Neurological Institute Screen for Higher Cerebral Functions.

**Table 4:** The neuronal injury, synaptic dysfunction and neurodegeneration blood-based biomarkers of PSCI.

Mechanism	Biomarker	Expression Change	Sample Size	Blood Fraction	Cognitive Test	Method	Evidence Level	Ref.
Neuronal Injury, Neurodegeneration and Synaptic Dysfunction Biomarkers	pNfL	Increase	1694	Plasma	MoCA	Simoa	Moderate	[12]
			264	Plasma	MoCA	Simoa		[185]
	pNfH	Increase	88	Serum	MoCA	ELISA	Low	[187]
	Tau	Decrease	55	Plasma	MoCA	IMR assay	Low	[188]
	p-Tau181	Increase	136	Plasma	MoCA	IMR assay	Low	[189]
	Aβ1-42	Decrease	55	Plasma	MoCA	IMR assay	High	[188]
			85	Plasma	MoCA and MMSE	IMR assay		[190]
			195	Serum	MoCA	ELISA		[191]
	GFAP	Increase	108	Plasma	MoCA	ELISA	Moderate	[93]
			336	Serum	MMSE	ELISA		[192]
	Neuroglobin	Decrease	353	Serum	MoCA	ELISA	Low	[193]
	BDNF	Decrease	660	Serum	MoCA	ELISA	Low	[194]
	NPTX2	Decrease	134	Serum	MoCA	ELISA	Low	[195]
	RA	Decrease	261	Serum	MoCA	ELISA	Low	[196]
	BACE1	Increase	152	Serum	MoCA	ELISA	Low	[197]
	sRAGE	Decrease	152	Serum	MoCA	ELISA	Low	[197]
	T3	Decrease	195	Serum	MoCA	CLIA	Low	[191]
	NPY	Increase	593	Plasma	MoCA	ELISA	Low	[198]

Abbreviations: pNfL, phosphorylated neurofilament light chain; pNfH, phosphorylated NfH; GFAP, glial fibrillary acidic protein; BDNF, brain-derived neurotrophic factor; NPTX2, neuronal pentraxin 2; RA, retinoic acid; BACE1, β-secretase enzyme; sRAGE, soluble receptor for advanced glycation end products; T3, triiodothyronine; NPY, neuropeptide Y; Simoa, single-molecule array; IMR, immunomagnetic reduction; CLIA, chemiluminescence immunoassay.

**Table 5:** The regulatory molecules and emerging blood-based biomarkers of PSCI.

Mechanism	Biomarker	Expression Change	Sample Size	Blood Fraction	Cognitive Test	Method	Evidence Level	Ref.
Regulatory Molecules And Emerging Biomarkers	miR-195	Increase	108	Serum	MoCA	RT-qPCR	Low	[229]
	miR-497	Increase	108	Serum	MoCA	RT-qPCR	Low	[229]
	miR-21	Decrease	77	Serum	MMSE	RT-qPCR	Low	[231]
	miR-511–3p	Decrease	169	Serum	MoCA	RT-qPCR	Low	[233]
	miR-93	Decrease	33	Plasma	Unknown	RT-qPCR	Low	[234]
	miR-let-7i	Increase	74	Serum	MoCA	RT-qPCR	Low	[235]
	miR-93-3p	Increase	172	Serum	MoCA	RT-qPCR	Low	[236]
	miR-502-3p	Increase	273	Serum	MoCA	RT-qPCR	Low	[237]
	SIX3OS1	Increase	138	Serum	MoCA	RT-qPCR	Low	[238]
	NEXN-AS1	Decrease	159	Serum	Unknown	RT-qPCR	Low	[239]
	hsa_circ_0089762	Increase	83	Plasma	MoCA	RT-qPCR	Low	[240]
	hsa_circ_0089763	Increase	83	Plasma	MoCA	RT-qPCR	Low	[240]
	hsa_circ_0064644	Increase	83	Plasma	MoCA	RT-qPCR	Low	[240]
	HDAC4	Decrease Or Increase	139	Whole blood	Unknown	RT-qPCR	Low	[241]

Abbreviations: miR-195, microRNA-195; HDAC4, histone deacetylase 4; RT-qPCR, real-time quantitative reverse transcription PCR.

**Table 6:** Clinical translation potential and evidence grading of representative blood-based biomarkers for PSCI.

Biomarker Category	Representative Biomarkers	Evidence Level	Validation Status	Clinical Applicability
Neuronal Injury, Neurodegeneration and Synaptic Dysfunction Biomarkers	Aβ1-42 PNfL GFAP	High	Increasing evidence from prospective cohorts; PSCI-specific external validation is still needed	Among the most promising candidates due to the biological relevance and improved detection sensitivity
Inflammation and Immune Biomarkers	IL-6 IL-8 RF HMGB1 NLR	Moderate-High	Associations reported in multiple cohorts; however, external validation remains limited	High accessibility, low cost, and easy clinical implementation; however, insufficient specificity limits independent clinical use
Oxidative Stress and Metabolic Biomarkers	Hcy FGF-21 UA	Moderate	Evidence is mainly from small observational cohorts with limited replication	Evidence is mainly from small observational cohorts with limited replication
Vascular Injury and Endothelial Dysfunction Biomarkers	MMP-9 Hb CysC FIB	Moderate	Supported by several studies, standardized protocols and multicenter validation are lacking	Provide additional information for PSCI risk stratification when combined with clinical characteristics and neuroimaging findings
Regulatory Molecules And Emerging Biomarkers	miR-195 miR-497	Low- Moderate	Mostly exploratory studies with limited independent replication	Provide novel insights into PSCI mechanisms and precision medicine approaches, but not currently ready for routine clinical use

## Data Availability

No new data were created or analyzed in this study. Data sharing is not applicable to this article.
